# Inferior vena cava hyperechoic structure in a patient with cardiac myxoma: a surgical case report and literature review

**DOI:** 10.1186/s44215-026-00277-y

**Published:** 2026-10-02

**Authors:** Hiroto Yasumura, Tomoyuki Matsuba, Ryusuke Ibi, Shinichi Minagoe, Atsuko Hiramine, Yuki Ogata, Koichiro Shimoishi, Yoshihiro Fukumoto, Goichi Yotsumoto, Yoshiharu Soga

**Affiliations:** 1 Department of Cardiovascular Surgery, Kagoshima City Hospital, 37-1, Uearatacho, Kagoshima City, Kagoshima 890-8760 Japan; 2 Department of Cardiovascular Medicine, Kagoshima City Hospital, Kagoshima, Japan; 3 Department of Cardiovascular Medicine, Nanpuh Hospital, Kagoshima, Japan; 4 Department of Cardiovascular Surgery, Graduate School of Medical and Dental Sciences, Kagoshima University, Kagoshima, Japan

**Keywords:** IVC, Thrombus, Pseudo-thrombus, Hyperechoic structure, Spontaneous Echo Contrast, SEC, Artifact, Mirror Image, Myxoma

## Abstract

**Background:**

IVC hyperechoic structure detected by echocardiography is relatively uncommon and is usually associated with deep vein thrombosis and cancers. In most cases, the etiology can be clarified using computed tomography (CT) and magnetic resonance imaging (MRI). However, we encountered an IVC hyperechoic structure that was not confirmed by multimodality imaging in a patient with cardiac myxoma.

**Case presentation:**

A 62-year-old woman presented with sudden-onset left hemiplegia and right homonymous hemianopsia. On arrival, her left arm was cold to the touch. Contrast-enhanced CT demonstrated a left atrial mass, left subclavian artery occlusion, and splenic infarction. Brain MRI revealed an acute infarction in the right occipital lobe. Transthoracic echocardiography (TTE) showed a mobile mass in the left atrium and a 33 mm immobile linear hyperechoic structure with surrounding blood flow in the suprarenal IVC, suggestive of organized thrombus. However, laboratory tests showed no elevation of D-dimer, and cardiac MRI did not confirm any IVC lesion. During surgical planning, there was concern that IVC cannulation via the right atrium for CPB might dislodge a potential thrombus and cause pulmonary embolism. Preoperative transesophageal echocardiography under general anesthesia demonstrated no IVC hyperechoic structure. An IVC cannula was inserted via the right femoral vein under fluoroscopic guidance. Following cardiac arrest and right atriotomy, direct inspection revealed no thrombus or other abnormal structure within the suprarenal IVC. The left atrial tumor was excised via left atriotomy. Histopathological examination confirmed cardiac myxoma. Postoperative TTE showed complete disappearance of the IVC hyperechoic structure.

**Conclusions:**

We report a rare case of IVC hyperechoic structure mimicking a true thrombus in a patient with cardiac myxoma. This phenomenon may be related with spontaneous echo contrast and echocardiographic artifacts. Our case highlights an important diagnostic pitfall in echocardiographic assessment of the IVC and emphasizes the importance of integrating findings from multiple imaging modalities.

**Supplementary Information:**

The online version contains supplementary material available at https://doi.org/10.1186/s44215-026-00277-y.

## Background

Cardiac myxoma is the most common primary benign cardiac tumor [1]. Surgical removal of cardiac myxoma requires cardiopulmonary bypass (CPB) with cannulation of the ascending aorta, superior vena cava (SVC) and inferior vena cava (IVC). Therefore, accurate preoperative assessment of the vena cava using multimodality imaging is essential for safe surgical planning.

IVC hyperechoic structure detected by echocardiography is relatively uncommon and is usually associated with deep vein thrombosis [2], hepatoma, renal cell carcinoma, and sarcoma of pelvic organs [3]. In most cases, the etiology can be clarified using other multimodality imaging, including computed tomography (CT) and magnetic resonance imaging (MRI). However, we encountered an IVC hyperechoic structure that was not confirmed by multimodality imaging in a patient with cardiac myxoma. We present this rare case with literature review.

## Case presentation

**Fig. 1:**
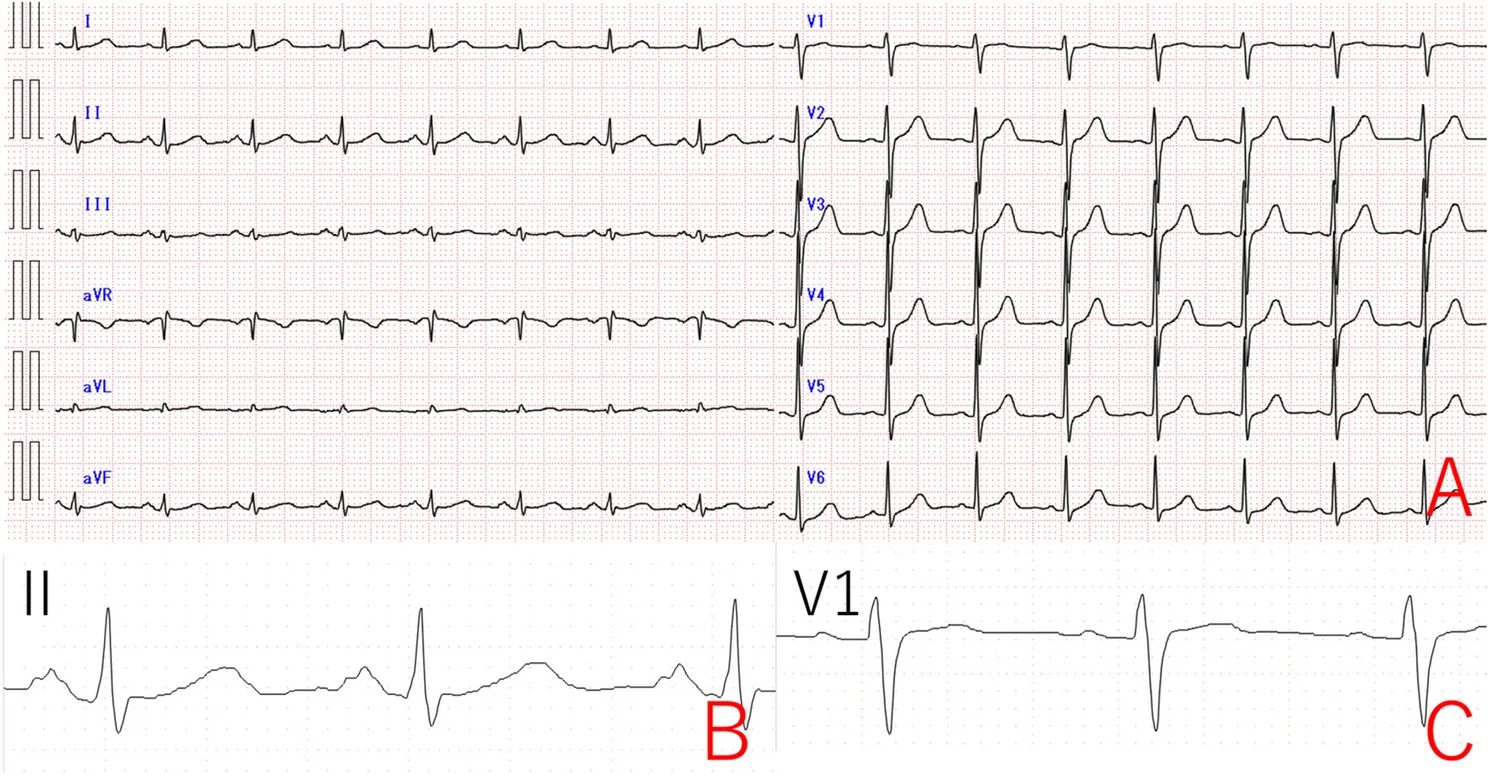
Preoperative 12-lead electrocardiographic (ECG) findings. **A** The ECG showed normal sinus rhythm with low voltage in precordial leads. **B** The ECG revealed an enhanced second hump in the P wave in lead Ⅱ. **C** The ECG did not show an enhanced negative deflection of the P wave in lead V1

**Fig. 2:**
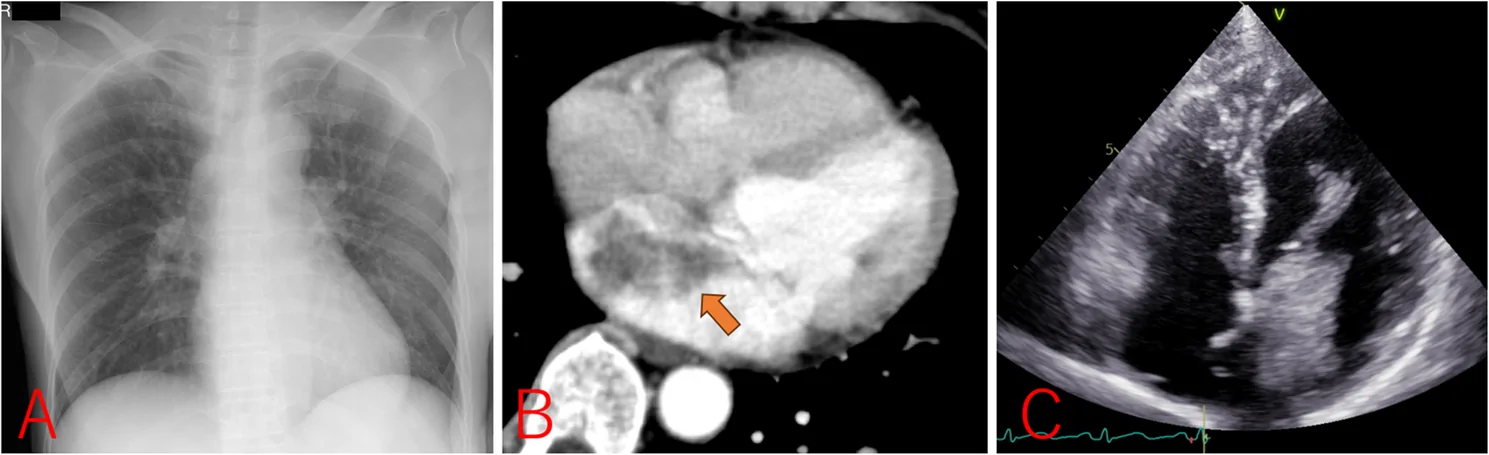
Preoperative imaging findings. **A** Chest radiography showed minimal pulmonary congestion. **B** Contrast-enhanced computed tomography demonstrated a left atrial tumor (orange arrow). **C** Transthoracic echocardiography showed a mobile, polypoid tumor in the left atrium

A 62-year-old Japanese woman　(height, 156.0 cm; weight, 48.2 kg) presented with sudden-onset left hemiplegia and right homonymous hemianopsia, and was transferred to our hospital. On arrival, her left arm was cold to the touch. Laboratory tests showed elevated white blood cell (14,800/µL), decreased hemoglobin (9.2 g/dL), elevated C-reactive protein (CRP) (7.02 mg/dL), hyperfibrinogenemia (624 mg/dL), and elevated D-dimer (4.7 µg/mL). Elevated CRP levels and hyperfibrinogenemia persisted, while subsequent D-dimer levels remained within the normal range. Twelve-lead electrocardiography (ECG) showed normal sinus rhythm (Fig. 1A).　The ECG also demonstrated an enhanced second hump in the P wave in lead Ⅱ (Fig. 1B), but did not show an enhanced negative deflection of the P wave in lead V1(Fig. 1C). Chest radiography showed minimal pulmonary congestion (Fig. 2A). Contrast-enhanced CT demonstrated a left atrial tumor(Fig. 2B, Video 1), occlusion of the left subclavian artery, and splenic and renal infarctions. Coronary CT revealed no stenosis of coronary artery. Brain MRI revealed an acute infarction in the right occipital lobe, and apixaban was subsequently initiated orally on hospital day (HD) 4 to prevent thromboembolic events. 

**Fig. 3:**
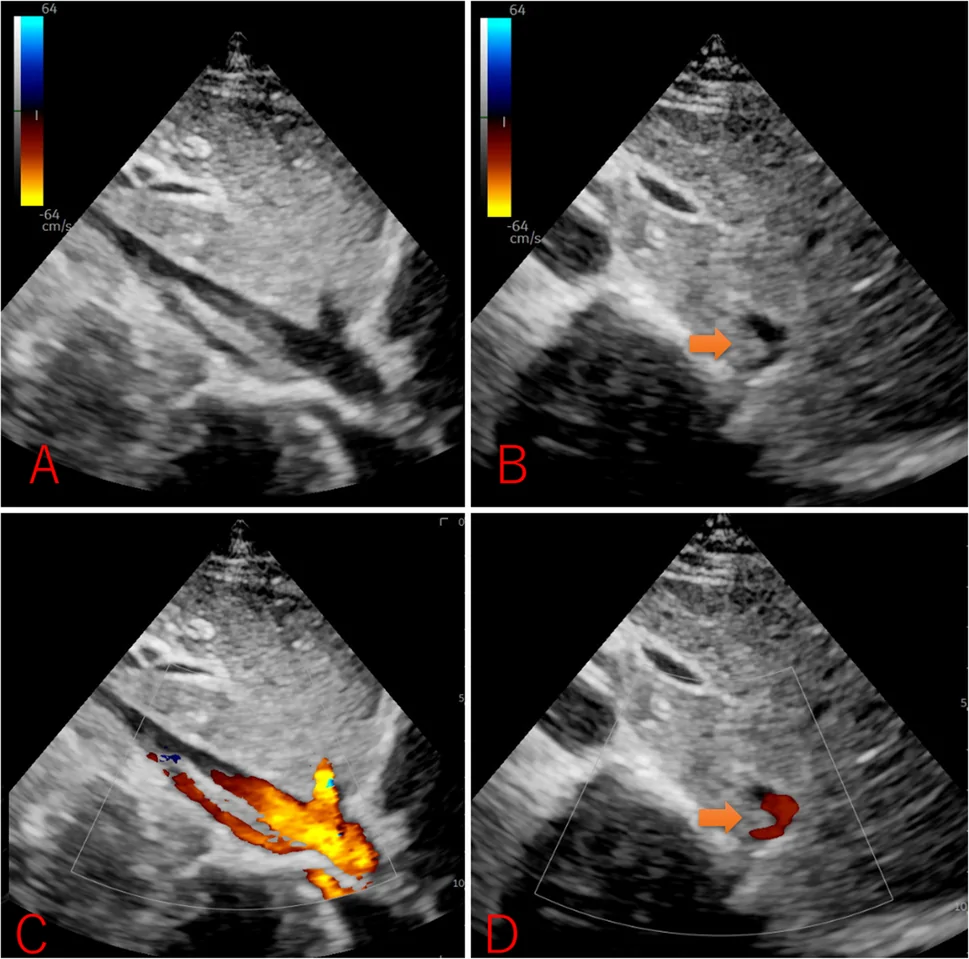
Preoperative findings of transthoracic echocardiography (TTE). **A** The long-axis view of TTE showed a 33mm, solitary, immobile, linear hyperechoic structure in the suprarenal inferior vena cava (IVC). **B** The short-axis view of TTE also showed a hyperechoic structure (orange arrow) in the suprarenal IVC. **C **The long-axis color Doppler view demonstrated blood flow surrounding the hyperechoic structure. **D **The short-axis color Doppler view also demonstrated blood flow surrounding the hyperechoic structure (orange arrow)

**Fig. 4:**
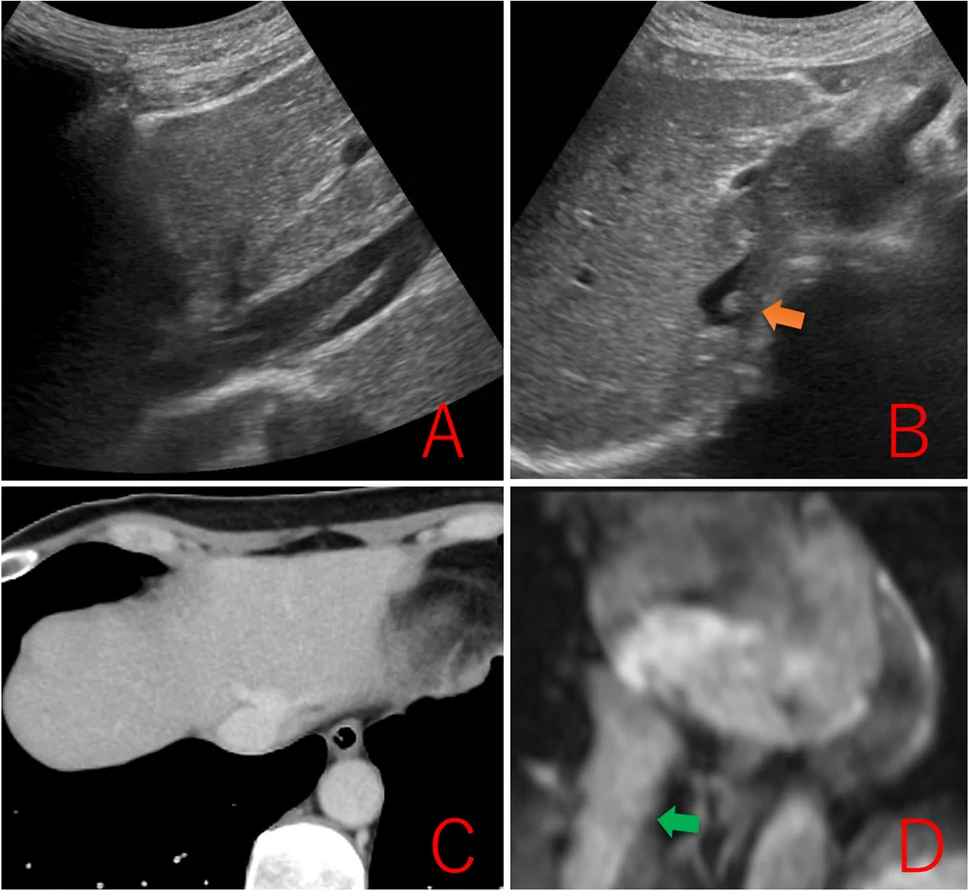
Preoperative findings of abdominal ultrasound, CT and MRI. **A** The long-axis view of abdominal ultrasonography (AUS) showed the same hyperechoic structure in the suprarenal IVC. **B** The short-axis view of AUS also showed a hyperechoic structure (orange arrow) in the suprarenal IVC. **C** Contrast-enhanced CT did not reveal any lesion in the IVC. **D** Cardiac MRI also did not demonstrate any IVC lesion (green arrow)

Transthoracic echocardiography (TTE) on HD 2 showed a large, mobile and polypoid tumor measuring 18 × 60 mm in the left atrium and its tail was moving back and forth between the left atrium and left ventricle in synchrony with ventricular contraction (Fig. 2C, Video 2). The diameter of left atrium, diastolic left ventricle, systolic left ventricle was 32 mm, 45 mm, and 32 mm, respectively. The ejection fraction of left ventricle was 55%. The regurgitation of aortic valve, mitral valve, and tricuspid valve was trace, mild, and trace, respectively. The second TTE performed on HD 18 revealed a 33-mm solitary, immobile, linear hyperechoic structure in the suprarenal IVC (Fig. 3A and B). Color Doppler imaging demonstrated blood flow surrounding the structure in both long- and short- axis views (Fig. 3C and D, Video 3, 4), suggesting an organized IVC thrombus. Abdominal ultrasonography (AUS) demonstrated the same IVC finding (Fig. 4A and B). TTE and AUS on HD 25 and 38 showed the same IVC findings. However, contrast-enhanced CT on admission (Fig. 4C) and cardiac MRI on HD 32 (Fig. 4D) did not reveal any corresponding lesion in the IVC.

**Fig. 5:**
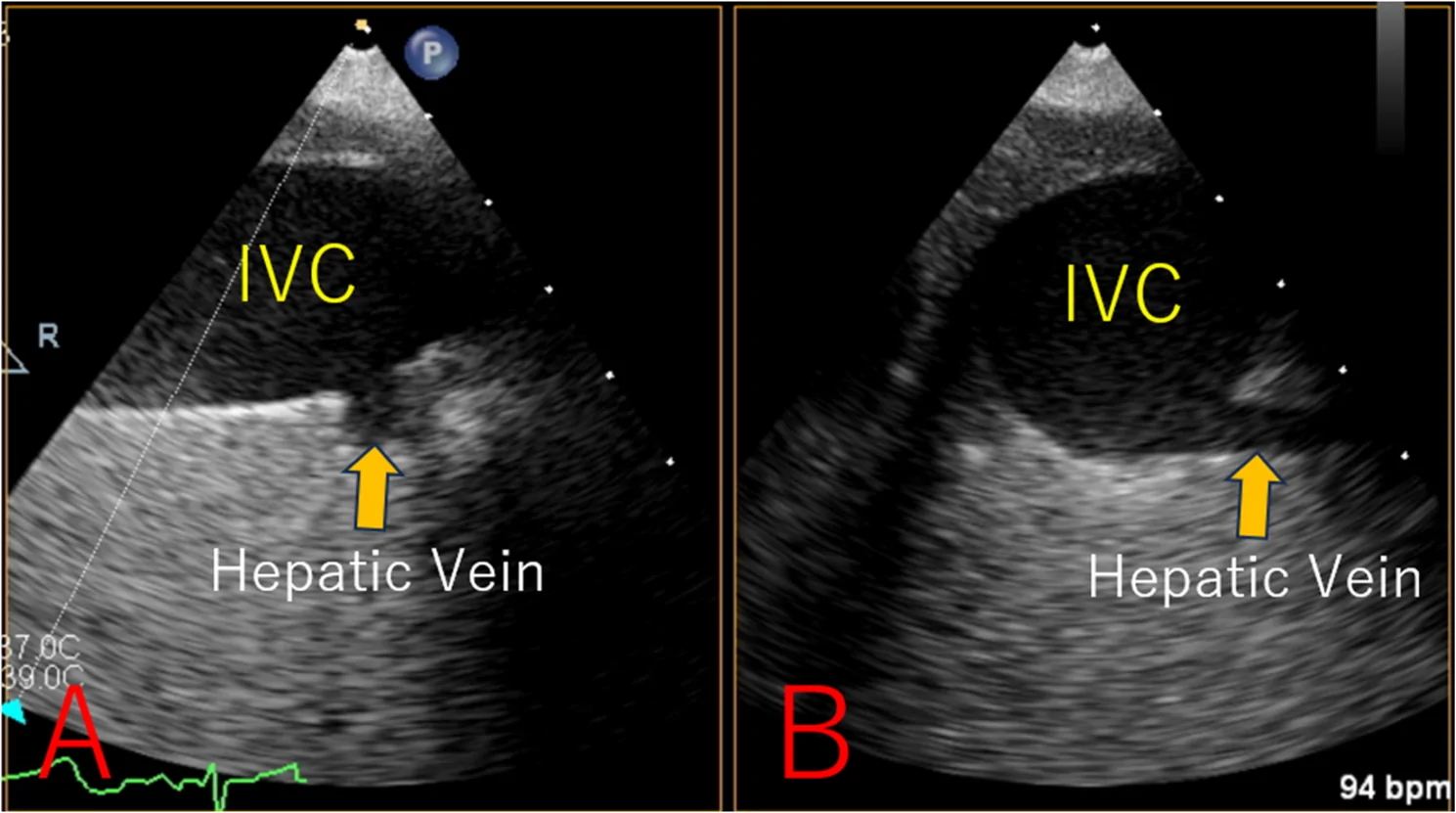
Preoperative and intraoperative findings on operation day. Transesophageal echocardiography showed no hyperechoic structure in the IVC in either long- axis (**A**) and short- axis (**B**) views

We performed tumor removal surgery under general anesthesia on HD 39. Under general anesthesia, hemodynamic assessment using a Swan Ganz catheter inserted via the right internal jugular vein showed a pulmonary artery pressure of 27/12 mmHg and a central venous pressure of 7 mmHg. Preoperative transesophageal echocardiography (TEE) under general anesthesia demonstrated no hyperechoic structure in the IVC in either long- or short- axis views (Fig. 5A). Following median sternotomy, CPB was initiated via cannulation of the ascending aorta and SVC. Given the concern that IVC cannulation via the right atrium for CPB might dislodge a potential thrombus and cause pulmonary embolism. Therefore, IVC cannula was inserted via the right femoral vein under fluoroscopic guidance. Following cardiac arrest and right atriotomy, direct inspection revealed neither thrombus nor abnormal anatomical structure such as a Chiari network or a prominent Eustachian valve within the suprarenal IVC and right atrium (Video 5). Subsequently, the left atrial tumor attached to the fossa ovalis was excised en bloc through a right-sided left atriotomy. The patient was successfully weaned from CPB, and the postoperative course was uneventful. Histopathological examination confirmed the diagnosis of cardiac myxoma. TTE on postoperative day (POD) 7 showed complete disappearance of the IVC hyperechoic structure. The patient was discharged on POD 22. Follow-up TTE on POD 204 demonstrated no recurrence of the myxoma and IVC hyperechoic structure.

## Discussion

Our case describes a rare instance of an apparent IVC hyperechoic structure detected on TTE and AUS in a patient with cardiac myxoma. To the best of our knowledge, an IVC hyperechoic structure associated with cardiac myxoma has not been previously reported. During the echography, the patient was placed in the spine position. The hyperechoic structure was suspected to represent a thrombus, but was not confirmed by contrast-enhanced CT, non-contrast MRI, nor preoperative TEE performed under general anesthesia. Contrasted-enhanced cardiac MRI, TEE performed before induction of general anesthesia, and TTE in the lateral decubitus position might have provided additional information regarding the IVC.

The IVC hyperechoic structure in our case was consistently observed in both long- and short- axis and was visualized on repeated TTE and AUS performed with different transducers, by different examiners, and on multiple days. These findings make a typical imaging artifact less likely [4]. However, direct intraoperative inspection failed to identify any intraluminal lesion. In addition, anticoagulation with apixaban was initiated on HD 4, whereas the D-dimer level was not elevated and the IVC hyperechoic structure was first described on HD 18. Taken together, these findings suggest that the observed structure was more likely to represent a pseudo-thrombus than a true thrombus.

Several mechanisms may explain the appearance of an IVC hyperechoic structure. One speculative and possible mechanism is spontaneous echo contrast (SEC) associated with low flow state and blood stasis [5, 6]. Although the precise mechanism of SEC remains incompletely understood, it may be attributed to aggregation of red blood cells mediated by fibrinogen under low-flow conditions [5, 7]. Its predisposing factors include atrial fibrillation, structural cardiovascular abnormalities, aging, low blood flow velocity, reduced shear rate, elevated erythrocyte sedimentation rate, hyperfibrinogenemia, and high hematocrit [5, 8, 9]. Several previous TTE-based case reports [5, 10] have described hyperechoic SEC within the IVC, particularly in patients with constrictive pericarditis, where it closely mimicked thrombus (Table 1). However, Meltzer et al. [11] evaluated 106 consecutive adults for SEC in the IVC using echocardiography and reported that SEC may also be observed in individuals without apparent pathology and may not be clinically significant. In our case, there was little clinical or hemodynamic evidence of significant venous stasis. Nevertheless, the SEC images reported by Hjemdahl-Monsen et al. [5] and Soma et al. [12] (Table 1) were similar to the findings in our case.

**Table 1:** Case reports describing spontaneous echo contrast (SEC) within the suprarenal IVC

Author	Published year	Age/Sex	Symptom assosiated with heart disease	Heart disease	LVEF	RVEF	IVC(mm)	Charecteristic of SEC	Surgery	Postoperative SEC
Hjemdahl-Monsen [5]	1984	66/M	Progressive fatigue	CP, Low cardiac output		Unknown		Moving toward the heart	Pericardiectomy	Resolved
Majumder [10]	2018	33/M	Progressive shortness of breath	CP, mild MR, mild TR	Normal	Normal	24	Very dense andslow moving	Pericardiectomy	Remained
Soma [12]	2024	20/F	Recurrent chest pain	None		Normal		• Moving toward the heart • Visible during both phases of the respiratory cycle	None	Remained
Present case	2026	62/F	None	Left atrial myxoma, mild MR	55%	Normal	16	33 mm-solitary, immobile, linear hyperechoic structure	Tumor removal	Resolved

*LVEF* left ventricular ejection fraction, *RVEF* right ventricular ejection fraction, *IVC* inferior vena cava, *SEC* spontaneous echo contrast, *CP* constrictive pericarditis, *MR* mitral regurgitation, *TR* tricuspid regurgitation

Another possible cause of an IVC hyperechoic structure is congenital abnormality. Normal anatomical variants, such as the Eustachian valve and Chiari network, can simulate thrombus in the right atrium or IVC [3, 13, 14]. Echocardiographic artifacts should also be considered, including side-lobe and beam-width artifacts, which can produce spurious intraluminal echoes and lead to false-positive findings [4, 15]. As far as we researched, no previous report has specifically described an IVC hyperechoic structure caused by these echogenic artifacts. Another potential artifact is the mirror- image artifact, which can produce an inverted image of the IVC at a deeper location or on the opposite side of the true IVC because of differences in the transit time of reflected ultrasound waves [16]. In such a case, the posterior wall of the true IVC between true and false IVC images may appear as a hyperechoic structure, potentially resembling a thrombus within a dilated IVC. In our case, an imaging-related pseudo-structure cannot be completely excluded, and artifact should therefore be considered an important differential diagnosis.

Because hemorrhagic transformation of a cerebral infarction before cardiac surgery requiring CPB may be life-threatening after the surgery, repeated brain MRI to determine the appropriate timing of the surgery. Hemorrhagic transformation may occur within 4 weeks after ischemic stroke [17], and cardiac surgery was therefore postponed until HD39 in our case. The careful selection of anticoagulant is also important. Compared with warfarin, apixaban has been associated with a significantly lower risk of major bleeding, particularly intracranial hemorrhage, while maintaining or improving protection against thromboembolic events [18]. Therefore, apixaban, initiated on HD 4, was considered unlikely to have contributed to the development of the IVC hyperechoic structure, which was first identified on HD 18, while its early initiation may have helped prevent hemorrhagic transformation of the cerebral infarction.

Our case highlights the importance of a careful differential diagnosis when an IVC hyperechoic structure is observed on TTE and AUS. Multimodality imaging, including contrast-enhanced CT, MRI, and TEE, is essential for establishing an accurate diagnosis. Based on our experience, if a similar case is encountered in the future, additional TTE in different patient positions and TEE performed before induction of general anesthesia should be considered preoperatively, as these approaches may provide additional information and help distinguish a true thrombus from a pseudo-thrombus. TEE has been reported to have a sensitivity and specificity of 68% and 98%, respectively, for the detection of intracardiac thrombi [19], and 97% and 99%, respectively, for the diagnosis of aortic dissection [20]. Integrating discrepant findings from multiple imaging modalities is therefore important.

## Conclusion

We report a rare case of IVC hyperechoic structure mimicking a true thrombus in a patient with cardiac myxoma. This phenomenon may be related with SEC and echocardiographic artifacts. Our case highlights an important diagnostic pitfall in echocardiographic assessment of the IVC and emphasizes the importance of integrating findings from multiple imaging modalities.

## Supplementary Information

Supplementary Material 1.

Supplementary Material 2.

Supplementary Material 3.

Supplementary Material 4.

Supplementary Material 5.

## Data Availability

The dataset supporting the conclusions of this article is included within the article.

## Abbreviations

CPB: cardiopulmonary bypass
SVC: superior vena cava
IVC: inferior vena cava
CT: computed tomography
MRI: magnetic resonance imaging
CRP: C-reactive protein
ECG: electrocardiography
HD: hospital day
TTE: transthoracic echocardiography
AUS: abdominal ultrasonography
TEE: transesophageal echocardiography
POD: postoperative day
SEC: spontaneous echo contrast
